# Assessment of degradability and endothelialization of modified poly L-lactic acid (PLLA) atrial septal defect (ASD) occluders over time in vivo

**DOI:** 10.1186/s13019-023-02401-3

**Published:** 2023-10-10

**Authors:** Jun Chen, Yumei Xie, Yifan Li, Xianmiao Chen, Mingjuan Fu, Yanfen Liu, Zhiwei Zhang

**Affiliations:** 1 The Second School of Clinical Medicine, Southern Medical University, Guangzhou, 510515 Guangdong China; 2 Department of Pediatric Cardiology, Guangdong Cardiovascular Institute, Guangdong Provincial People’s Hospital (Guangdong Academy of Medical Sciences), Southern Medical University, Guangdong Provincial Key Laboratory of South China Structural Heart Disease, No. 106 Zhongshan Second Road, Yuexiu District, Guangzhou, 510100 Guangdong China; 3 Danzhou People’s Hospital, Danzhou, 571700 Hainan China; 4 Lifetech Scientific (Shenzhen) Co., Ltd., Shenzhen, 518057 Guangdong China

**Keywords:** Atrial septal defect, Poly L-lactic acid, Occluder, Degradability, Endothelialization, Congenital heart disease

## Abstract

**Objective:**

To evaluate the fiber-degradation and endothelialization of a modified poly L-lactic acid (PLLA) atrial septal defect (ASD) occluder for a long time in vivo.

**Methods:**

A total of 57 New Zealand rabbits were selected to establish the vasculature implantation model, which would be used to characterize the mechanical properties and pathological reaction of PLLA filaments (a raw polymer of ASD occluder). In total, 27 Experimental piglets were used to create the ASD model for the catheter implantation of PLLA ASD occluders. Then, X-ray imaging, transthoracic echocardiography, histopathology, and scanning electron microscope (SEM) were performed in the experimental animals at 3, 6, 12, and 24 months after implantation.

**Results:**

In the rabbit models, the fibrocystic grade was 0 and the inflammatory response was grade 2 at 6 months after vasculature implantation of the PLLA filaments. The mass loss of PLLA filaments increased appreciably with the increasing duration of implantation, but their mechanical strength was decreased without broken. In the porcine models, the cardiac gross anatomy showed that all PLLA ASD occluders were stable in the interatrial septum without any vegetation or thrombus formation. At 24 months, the occluders had been embedded into endogenous host tissue nearly. Pathological observations suggested that the occluders degraded gradually without complications at different periods. SEM showed that the occluders were endothelialized completely and essentially became an integral part of the body over time.

**Conclusion:**

In the animal model, the modified PLLA ASD occluders exhibited good degradability and endothelialization in this long-term follow-up study.

## Introduction

Atrial septal defect (ASD) accounts for up to 30% of patients with congenital heart disease (CHD), and interventional treatment has been considered a preferred option for it [1]. Currently, many kinds of occluders have been widely used in ASD treatment. Most occluders were made by hyperelastic nickel-titanium-alloy, but non-degradable, which perhaps accompanied by many complications such as erosion of the device, delayed endothelialization, and thrombus formation [2–4]. The ideal occluder is the occluder implanted to form the host tissue, and the materials of the occluder in the host tissue are degraded and absorbed. Thus, the ideal occluder will eventually disappear, completely replaced by the host tissue. The biodegradable occluder may avoid long-term complications due to the permanent presence of non-degradable materials within the host tissue. There is no available ASD occluder made of fully biodegradable polymer biomaterials.

The development and preparation of high molecular polymer biodegradable stents have aroused increasing attention. Polymers have long been used in the biomedical engineering field as fully biodegradable polymer materials, like poly D, L-lactide (PDLLA), poly L-lactide (PLLA), poly trimethylene carbonate (PTMC), etc. Moreover, their low biotoxicity and immunity have been confirmed too [5–8]. In particular, PLLA could finally degrade into carbon dioxide and water in vivo, and have been approved for clinical use by the Food and Drug Administration (FDA) since 1997 [9]. PLLA could even be used with metal devices such as iron coronary stents to tune their biodegradation rates [10]. PLLA has been widely used in orthopedics, holistic surgery, and vascular medicine. It possesses excellent mechanical properties, high strength and elastic modulus, and good supporting force. The degradation period of PLLA is suitable, and the maintenance time of effective support is greater than 6 months.

In the present article, PLLA materials were used to fabricate an ideal ASD occluder. In 2019, we demonstrated the differences between biodegradable occluders and conventional Nitinol occluders in short-term follow-up points, including gross anatomy, ultrasound follow-up, and scanning electron microscopy (SEM) observation of tissue growth on the surface of the instrument, but did not focus on the degradation and endothelalization process of the instrument [11]. In this paper, the degradation rate and local tissue response of PLLA in vivo were investigated in detail by PLLA filament implantation in rabbit abdominal aorta. The endothelialization processes of different parts on the surface of PLLA occluders were detected by SEM and immunohistochemistry (IHC). And the long-term follow-up results were discussed to investigate the efficiency and safety of the device.

## Materials and methods

### Fabrication of PLLA ASD occluder

**Fig. 1:**
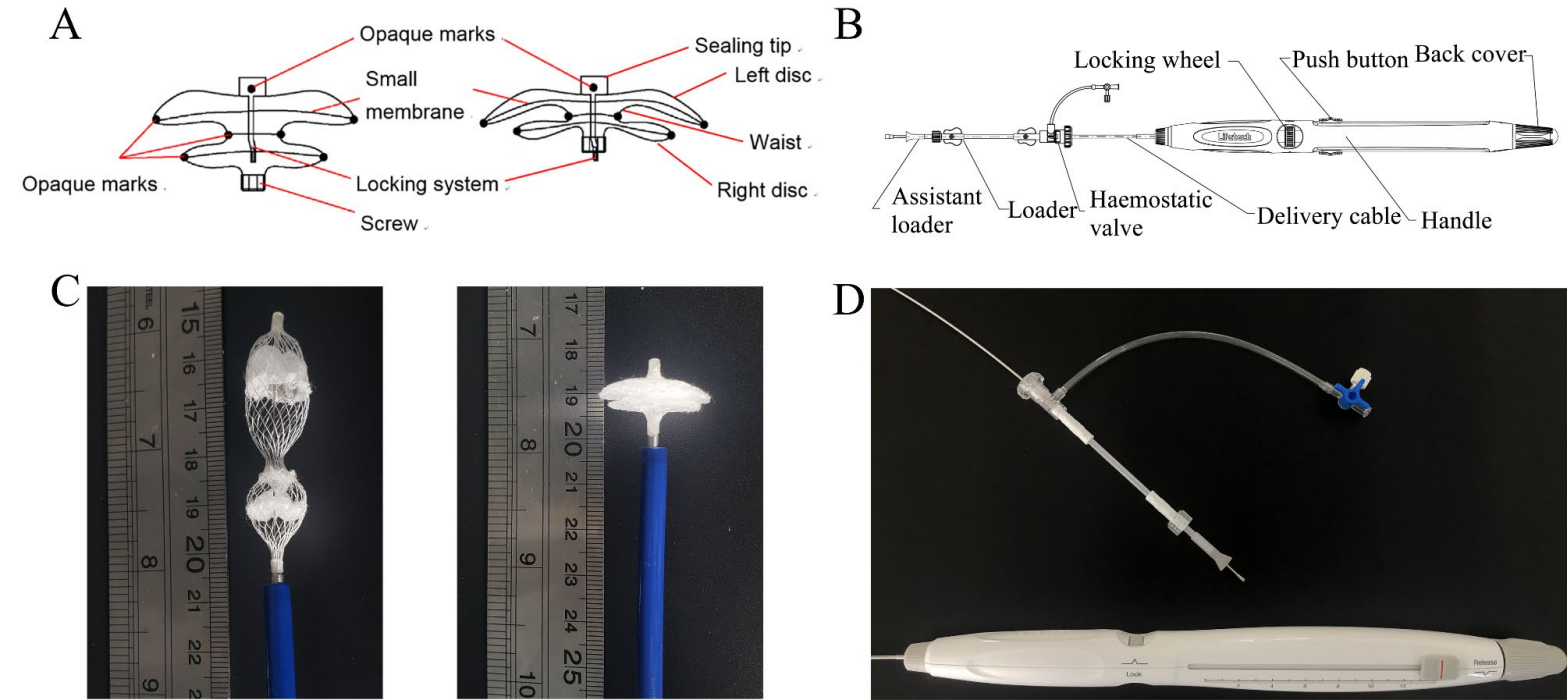
The PLLA device and the delivery system. (**A**) Schematic drawing of the PLLA occluders; (**B**) Schematic drawing of the delivery system; (**C**) The PLLA occluder device; (**D**) The delivery system. PLLA: poly L-lactide

We designed and made a modified ASD occluder suitable for percutaneous ASD closure (Fig. 1A-D). This occluder has a self-expandable double umbrella framework, and the two-disc-like part is made of 0.15 mm PLLA filaments by monofilament woven and thermoforming technology. The occluder uses two kinds of molecular weight PLLA filament, and the characteristic viscosity is 1.6 dL / g and 1.0 dL/g, respectively. The characteristic viscosity corresponds to the average adhesive molecular weight of the polymer material. Biodegradable PLLA membranes were placed into two umbrellas and the waist to enhance the closure effect by promoting thrombosis and endothelial formation. The PLLA material does not show up on radiographs, and 7 platinum-iridium spots are attached to the device as radio-opaque markers. Moreover, X-ray imaging can only display 7 platinum-iridium spots of the PLLA occluder, used to trace the occluder during surgery. Besides, PLLA material can be imaged by ultrasound. Therefore, X-ray imaging and ultrasound were utilized to observe the position and release development of the PLLA occluder. The specifications of the occluder range from 06 to 32 mm, with a 2 mm interval between adjacent specifications. The specifications of the occluder are defined by the waist diameter.

### Animal model generation

The safety and effectiveness of PLLA occluder were evaluated by observing adverse events such as shunt and thrombosis, local and systemic inflammation indicators, endothelization, and degradation of occluder gross anatomy after implantation in the pig atrial septal defect animal model. However, it was impossible to observe the degradation mechanical changes, mass changes, and molecular weight changes of PLLA silk after implantation in the simulated instrument environment. The degradation mechanical changes, molecular weight changes, and mass changes of PLLA silk could be observed when PLLA filament was implanted in the rabbit abdominal aorta model. The combination of the two models can better observe the safety and effectiveness of the occluder.

#### Model of PLLA filaments implanted in New Zealand rabbits’ abdominal aorta

**Fig. 2:**
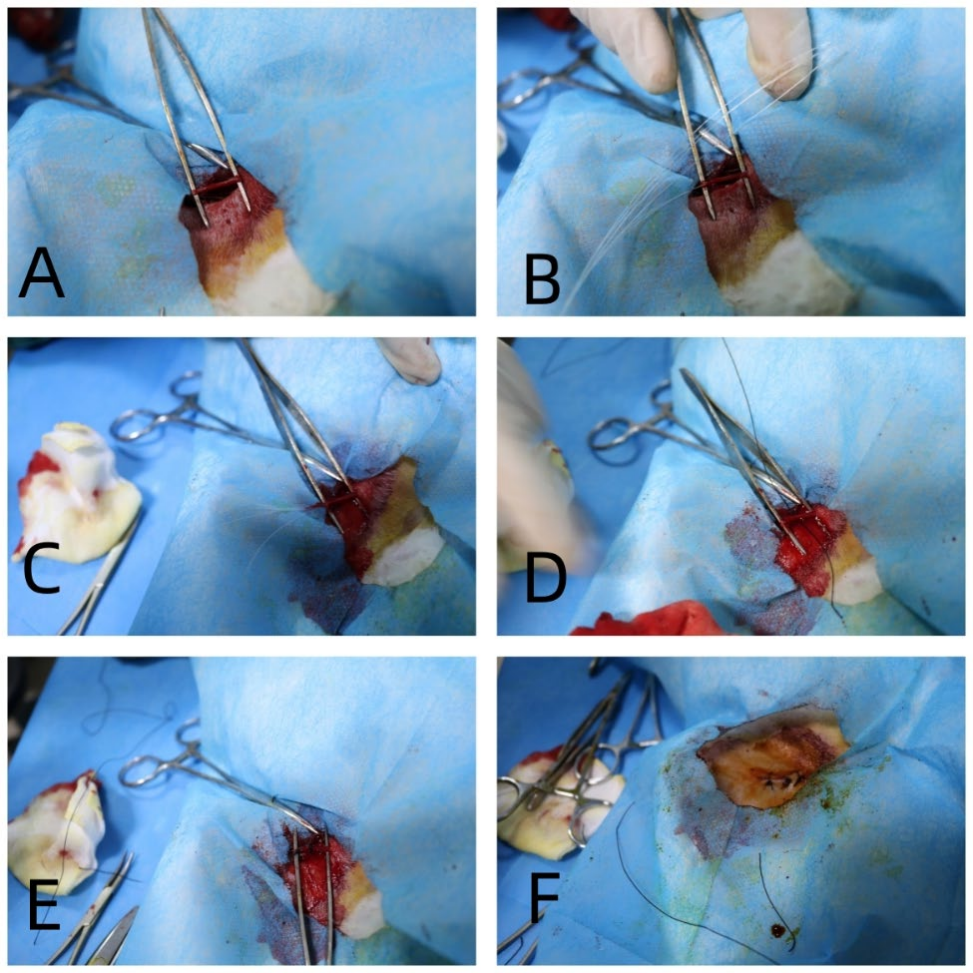
Steps of PLLA filaments implanted in rabbits’ abdominal aorta. (**A**) Free proximal femoral artery was conducted; (**B**, **C**) The femoral artery was incised and PLLA wire was implanted from the femoral artery into the abdominal aorta; (**D**, **E**) Femoral artery ligation was performed; (**F**) The wound was sutured. PLLA: poly L-lactide

New Zealand rabbits were selected to establish the vasculature implantation model. All rabbit experiments were performed under the anesthesia of intravenous injection of pentobarbital sodium. The right/left femoral artery was selected to be incised. Six PLLA filament samples of the same size were implanted into the aortaventralis along the blood vessels, and sent below the opening of the left and right renal arteries. Six PLLA filament samples of the same size were implanted into the aortaventralis of each animal. Standard surgical procedure sutures the wound and completes material implantation. The implanted PLLA filament material was the same as that used in the PLLA occluder. The implant sizes were NO.1021, NO.1023, and NO.1627. NO.1021 refers to PLLA filament with characteristic viscosity of 1.0 dL/g and diameter of 0.21 mm. A total of 57 rabbits were successfully implanted with PLLA filament. The brief steps are shown in Fig. 2A-F.

#### Model of biodegradable PLLA ASD occluder implanted into piglet ASD

**Fig. 3:**
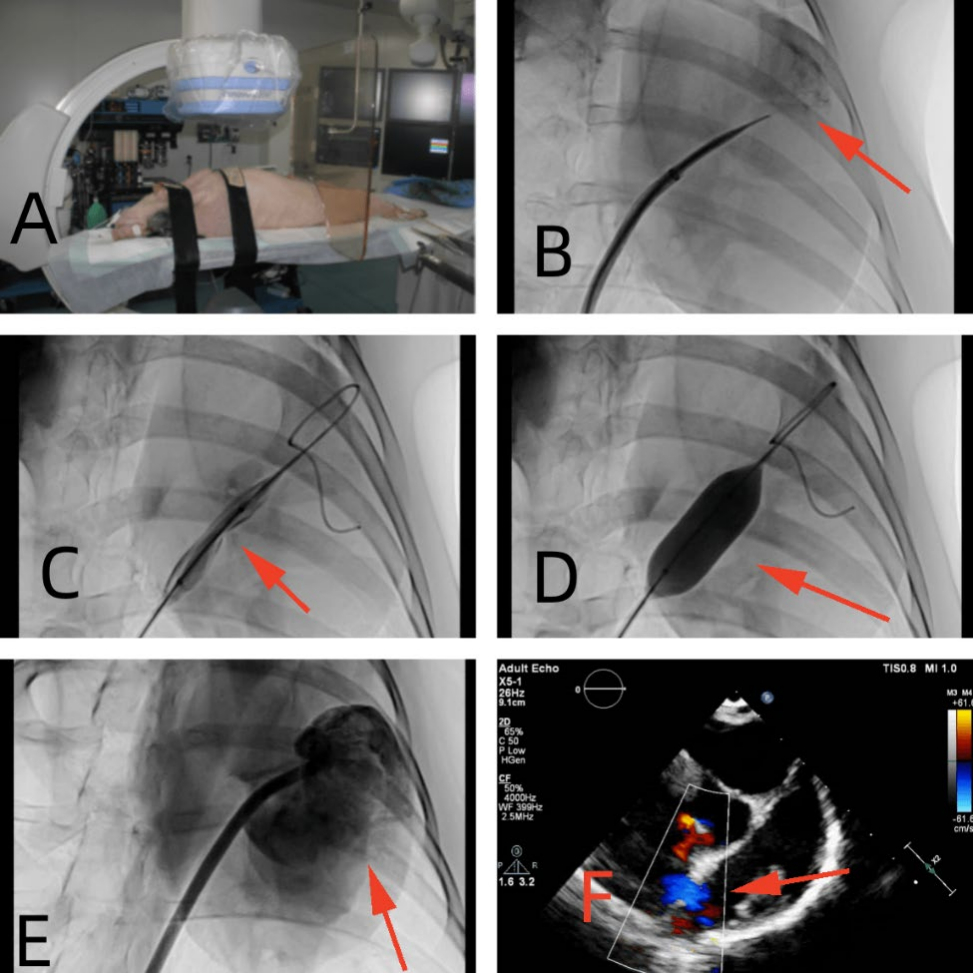
Steps of pigs implanted with PLLA occluders. (**A**) Pigs were given tracheal intubation under general anesthesia; the red arrow indicated the atrial septum. (**B**) Atrial septal puncture; the red arrow indicated the Left atrium. (**C**, **D**) The balloon dilates the atrial septum; the red arrow indicated the atrial septum. (**E**) Left atrial angiography; the red arrow indicated the Left atrium. (**F**) Ultrasonic measurement of atrial septal defect; the red arrow indicated the ASD. PLLA: poly L-lactide. ASD: atrial septal defect

**Fig. 4:**
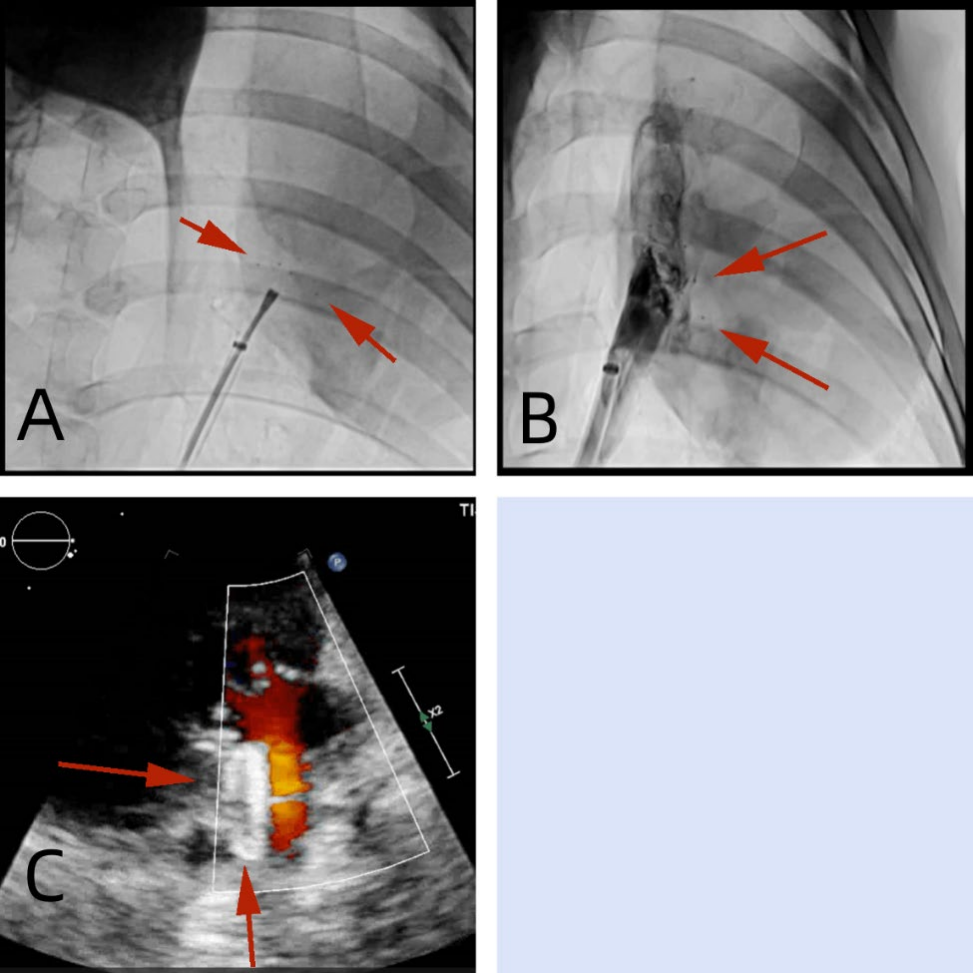
Imaging examination of pigs implanted with PLLA occluders. (**A**) The PLLA occluder was released through the X-ray position of seven platinum-iridium markers; (**B**) X-ray after release of PLLA occluder; (**C**) Ultrasonic morphology and position after release of PLLA occluder. Red arrows indicated the PLLA ASD occluder. PLLA: poly L-lactide. ASD: atrial septal defect

Experimental piglets were used to establish an ASD model for biodegradable PLLA ASD occluder implantation. Before the operation, all animals underwent clinical examination and transthoracic echocardiography (TTE) scanning to demonstrate that they were healthy with morphologically normal hearts. All animal experiments were performed with intubation ventilation under general anesthesia. Through the femoral vein, we perforated the atrial septum using an atrial septal puncture technique, then expanded the atrial septum with a 12–15 mm balloon to create a model of the atrial septal defect, and implanted a PLLA occluder. The details of the procedure have been previously described [12]. A total of 27 pigs were successfully implanted with PLLA occluders. The brief steps are shown in Figs. 3A-F and 4A-C.

All animals were obtained from Mingzhu Experimental Animal Scientific Technology Company (Dongguan City, Guangdong Province, China). The research was approved by the Research Ethics Committee of Guangdong General Hospital/Guangdong Academy of Medical Sciences.

### Mechanical properties and pathological study of PLLA filament after intravascular implantation

New Zealand rabbits implanted with PLLA filament were raised normally. Samples implanted in each rabbit were weighed and recorded before being cleaned and sterilized. Three rabbits of each kind of silk were selected at each time node in January, February, April, and June after surgery, then sacrificed. All PLLA filament plant samples of each rabbit were removed. Two filament fibers were taken for mechanical property test, and the remaining filament was tested by Gel permeation chromatography (GPC), which gave the molecular weight and residual mass. When the residual mass was measured by GPC, the initial mass of the GPC sample was set as the pre-implantation PLLA filament mass minus the mechanical properties test sample mass. Six months after the operation, 3 rabbits of each silk type were sacrificed, and all PLLA filament plant samples of each rabbit were removed. Then, slices were sliced and stained for pathological examination.

### Follow-up studies

Follow-ups of piglets after implantation were done to evaluate long-term complications. The contents included daily monitoring of appetite, mental status, physical condition, daily routine, bowel movements, urination, and neurological manifestations (especially dystrophy and hemiplegia). In addition to the preoperative examination, TTE and blood testing were conducted for every 2–3 animals at 3, 6, 12, and 24 months after implantation, respectively, and then they were sacrificed, with the hearts removed. Baseline and follow-up assessment was geared at evaluating the effectiveness and safety of the occluders by documenting nutritional status, inflammatory status, coagulation function, thrombosis, occluder location and shape, and cardiac structure and function. Occluder location and shape and adjacent anatomic structures were evaluated by gross anatomical examination. The tissues used for HE staining and IHC were fixed with 4% paraformaldehyde (PFA). After 4% PFA fixation for 24 h, paraffin slice wax blocks were prepared. The tissue used for electron microscope examination was fixed with electron microscope fixation solution. Hematoxylin-eosin (HE) staining, IHC, and electron microscopy were used to assess the hyperplasia of tissues surrounding occluders and PLLA degradation.

### HE staining

The thickness of the paraffin section was 3 μm. The tissue section was baked in the oven (65℃) for 4 h, dewaxed using xylene, and then rinsed with running water. The sections were stained using Mayer’s hematoxylin solution for 5 min, differentiated with 0.5% hydrochloric acid alcohol for 2 s. Then, the slices were stained using alcohol-soluble eosin for 3 min, dehydrated by alcohol, treated with xylene, and sealed by neutral resin finally. Images were captured using the digital tissue biopsy scanner (Hungary 3D AISTECH, Pannoramic SCAN).

### IHC

The thickness of the paraffin section was 3 μm. The tissue section was baked in an oven (65℃) for 4 h, then dewaxed using xylene. Alcohol was used to remove xylene. The antigen repair was conducted with 0.01 mol/L citrate buffer (PH 6.0) under microwave medium fire for 10 min. Then, the slices were treated with 0.3% hydrogen peroxide for 10 min. 1% fetal bovine serum was used to block nonspecific antigenic sites for 15 min. Excess serum was removed and the anti-CD31 antibody (Abcam, ab28364, 1:50) was added and incubated at 4℃ overnight. The corresponding secondary antibody (Abcam, ab288151, 1:500) was added to the slices and incubated at room temperature for 1 h. DAB was used for color development for 3 min. The slices were rinsed with running water to stop the reaction. The nucleuses were stained using Mayer’s hematoxylin for 5 min. We used neutral gum for sealing. Images were captured using the digital tissue biopsy scanner (Hungary 3D AISTECH, Pannoramic SCAN).

### Examination of SEM

Samples fixed with 2.5% glutaraldehyde for 48 h were rinsed with phosphate buffer, and then fixed with 1% osmic acid for 1.5 h. Alcohol gradient dehydration was conducted, followed by isoamyl acetate treatment. The samples were placed close to the conductive carbon film double-sided tape, and then placed on the ion sputter sample table for gold spraying for about 30 s to conduct conductive treatment of the sample. We adopted SEM (Hitachi, SU8100) to observe the target area, and captured images.

## Results

### Mechanical property of PLLA filament endovascular implantation

**Fig. 5:**
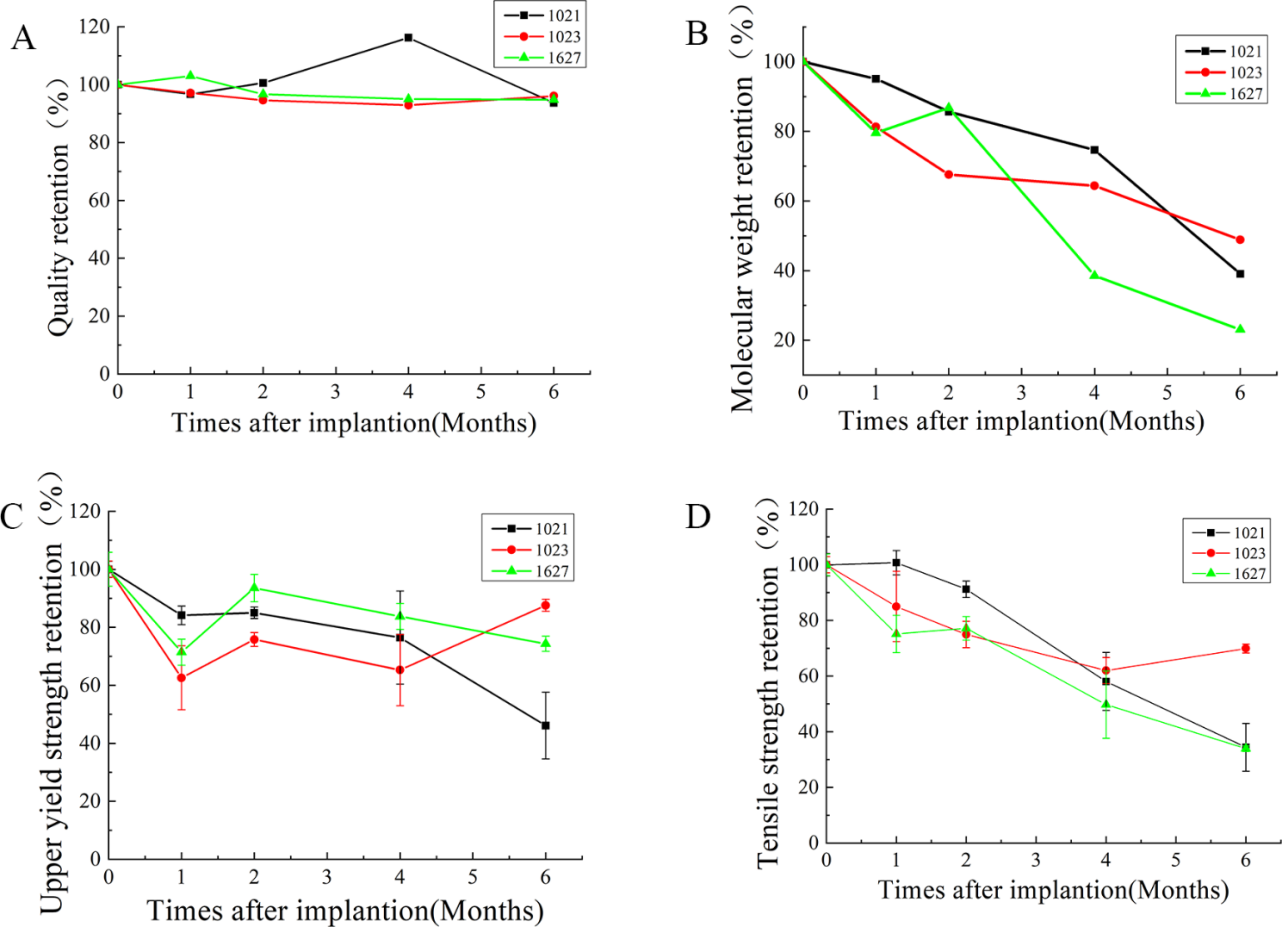
Detection of Mechanical property of PLLA filaments after endovascular implantation. (**A**) Quality retention; (**B**) Molecular weight retention; (**C**) Upper yield strength retention; (**D**) Tensile strength retention. PLLA: poly L-lactide

For simulating the device application environment, the PLLA filaments were implanted into the vasculature to monitor the changes in mechanical strength or molecular weight of PLLA. Quality retention (Fig. 5A): The mass loss rates were not significant for all PLLA filaments at 0 to 6 months after endovascular implantation. That means the degradation of the PLLA filament only produced a few soluble small molecules after 6 months in vivo. Molecular weight retention (Fig. 5B): The molecular weight of the PLLA filament decreased gradually with the increase of implantation time. The molecular weight retention rate was higher than 20% at 6 months of implantation, indicating that molecular chains of PLLA filaments were broken during degradation. Mechanical strength retention (Fig. 5C, D): The upper yield strength remained stable at 40% above, and the tensile strength kept up at 30% of all PLLA filaments at 6 months after endovascular implantation. At this point, the risk of filament breakage could be negligible, because the PLLA filaments have been endothelized after 6 months.

### Pathological analysis of PLLA filament endovascular implantation

**Fig. 6:**
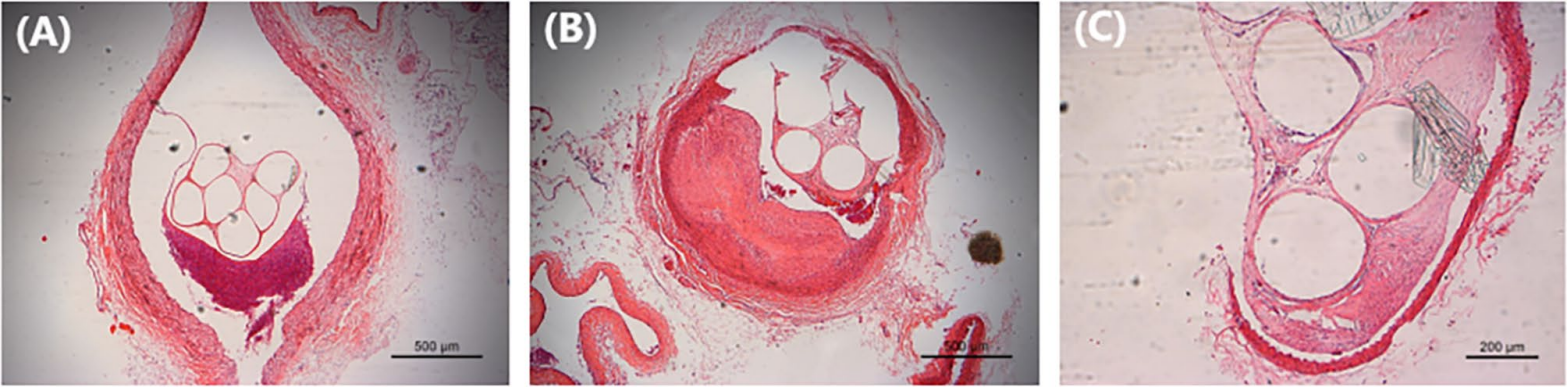
Pathological observation of PLLA filaments at 6 months after endovascular implantation. (**A**) PLLA filament of NO.1021; (**B**) PLLA filament of NO.1023; (**C**) PLLA filament of NO.1627. PLLA: poly L-lactide

For simulating the device application environment, the PLLA filaments were implanted into the vasculature to observe the effect of the PLLA on local tissues. As shown in Fig. 6(A-C), at 6 months after the implantation of PLLA filaments (labeled: NO.1021, NO.1023, and NO.1627), a few lymphocytes were found around them, with inflammatory response grade 2. The fibrocystic grade of NO.1021 and NO.1023 PLLA filaments was 0, which is the PLLA with a thin cystic and a few fibrocytes only. The fibrocystic grade of NO.1627 was I, which is the PLLA with a small number of collagen fibroblasts and fibroblasts occasionally.

### Followup studies

**Fig. 7:**
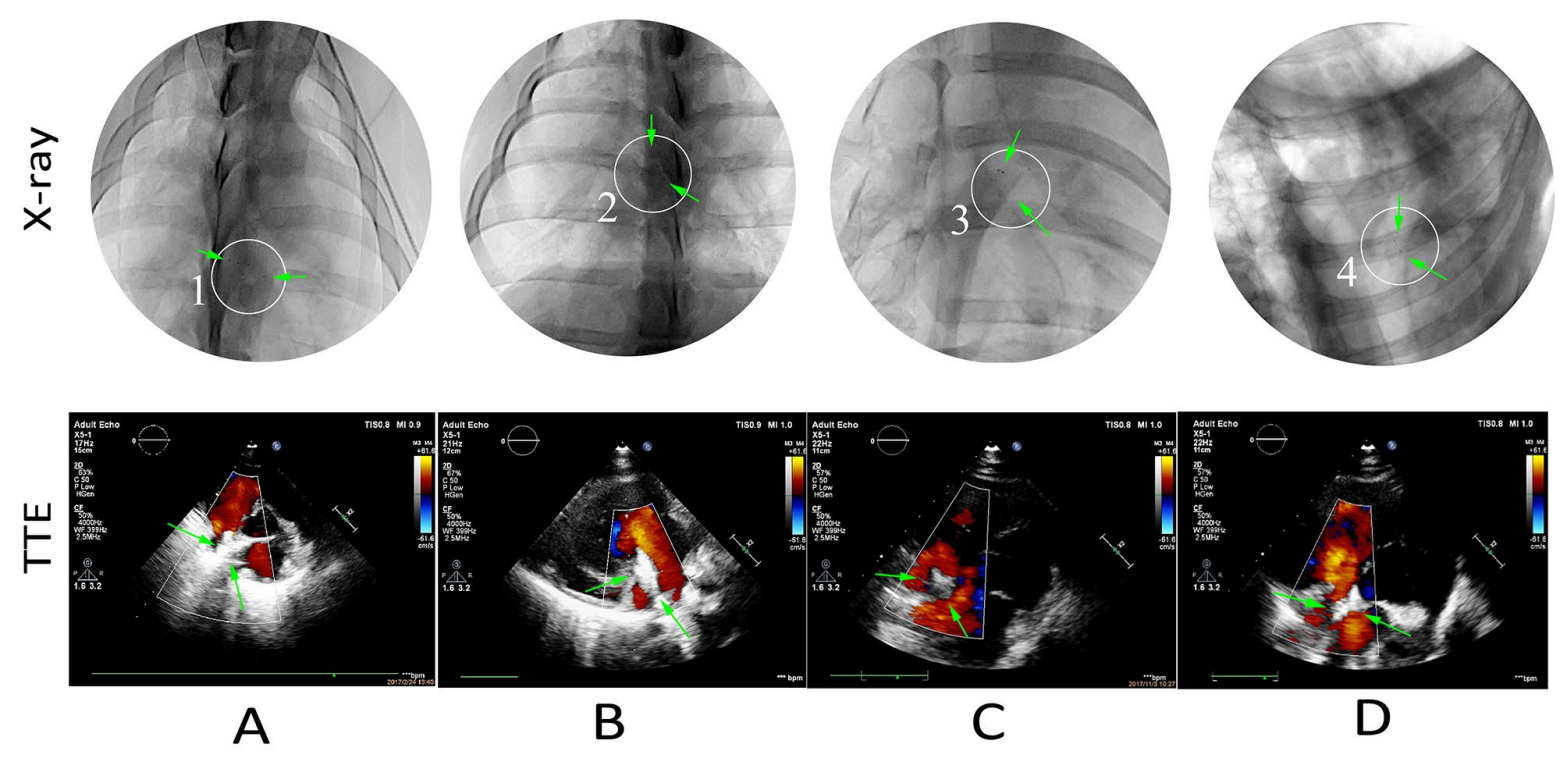
Postoperative examinations of PLLA occluders by X-ray and transthoracic echocardiography (TTE). (**A**) 3 months after implantation. (**B**) 6 months after implantation. (**C**) 2 months after implantation. (**D**) 24 months after implantation. Green arrows indicated the PLLA ASD occluder. PLLA: poly L-lactide.ASD: atrial septal defect

All animals survived in well general state, with good appetite, free movement, and normal behavior, and without infection, dyspnea, or hematochezia. At 3, 6, 12, and 24 months after implantation, X-ray fluoroscopy confirmed that there was no shift in implanted PLLA ASD occluder (Fig. 7A-D). TTE examination reported that there was no residual shunting, thrombosis, pericardial effusion, mitral regurgitation, tricuspid regurgitation, or pulmonary vein obstruction after device implantation (Fig. 7A-D).

### Macroscopic examination of cardiac anatomy

**Fig. 8:**
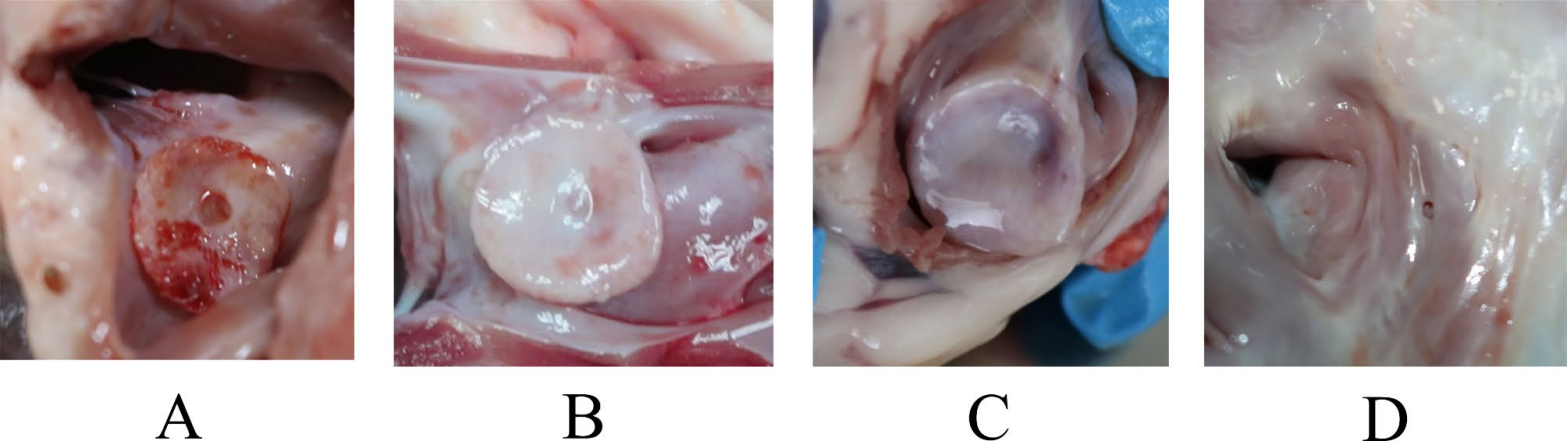
Macroscopic examination of PLLA ASD occluder at different time points after implantation. (**A**) 3 months after implantation. (**B**) 6 months after implantation. (**C**) 12 months after implantation. (**D**) 24 months after implantation. PLLA: poly L-lactide.ASD: atrial septal defect

All occluders resided in the interatrial septum of the cardiac anatomy, which did not destroy the morphology of the heart, the volume of the heart cavity, the thickness of the myocardial wall and blood vessels. Besides, the occluders could not cause vegetation and thrombusis. At 3 months, the PLLA ASD occluder retained the original shape, and was covered with a layer of milky white film (Fig. 8A). At 6 months, the surface of the PLLA ASD occluder was overcovered with a newborn film mixed with a little red, and its shape was still clearly visible (Fig. 8B). At 12 months, PLLA ASD occluder was overcovered with a reddish-white film, and partially fused with the endocardial tissue (Fig. 8C). At 24 months, the PLLA ASD occluder was mostly fused with the endocardial tissue, and the shape of the occluder was obscure (Fig. 8D). At every time point, no abnormalities in the gross specimens of the lung, liver, spleen, and kidneys were noted, and no infarction was observed by naked-eye examination.

### Histopathological examination of occluder degradation

**Fig. 9:**
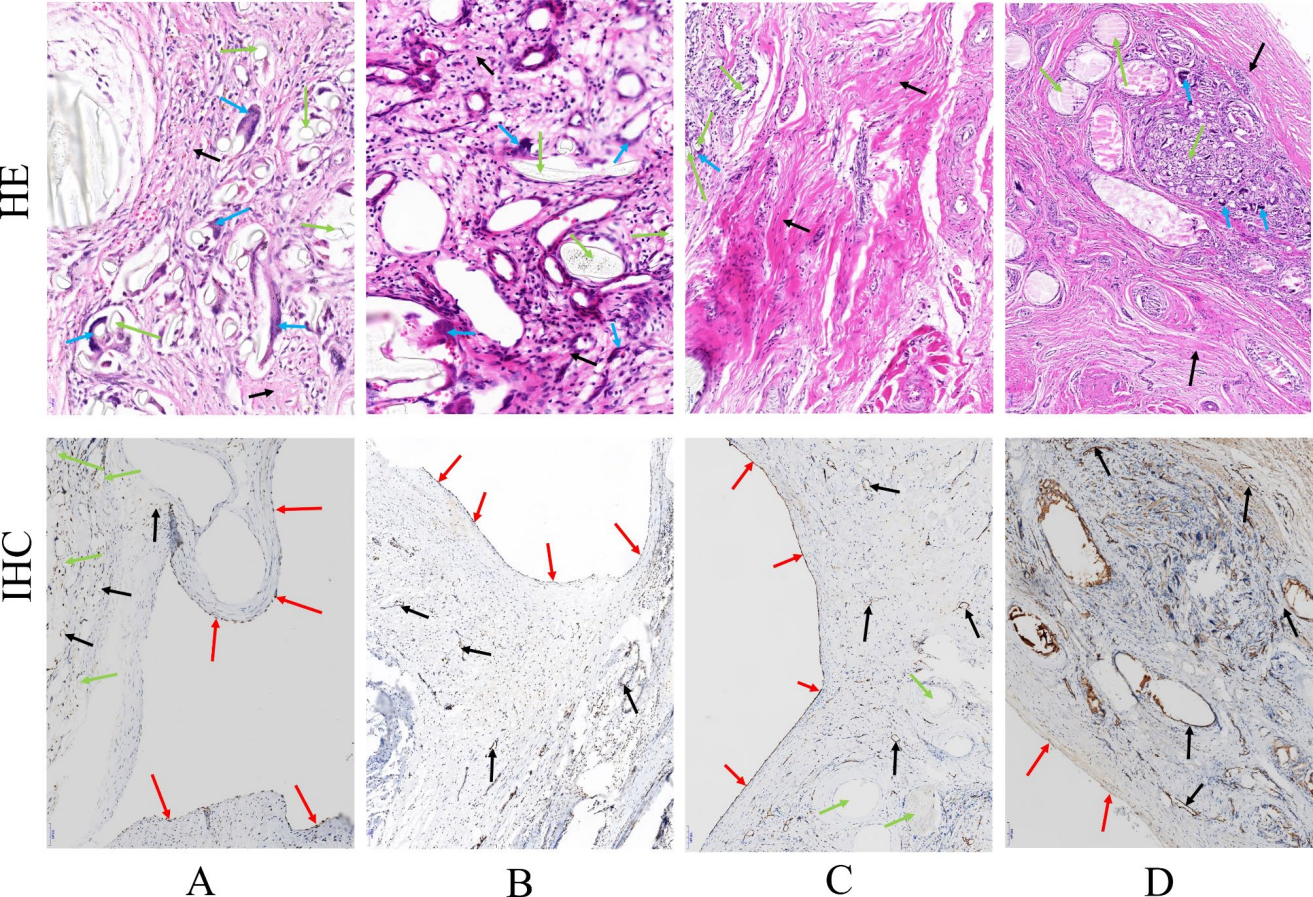
Histopathological observation of PLLA ASD occluders by HE staining and IHC at different follow-up points of 3 (**A**), 6 (**B**), 12 (**C**), 24 (**D**) months. Green arrows indicated the PLLA ASD occluder. Blue arrows indicated fibroblasts, collagen fibers, inflammatory cells or multinuclear giant cells. Red arrows indicated the endothelial cells in the new endocardium. Black arrows indicated the endothelial cells in new small vessels. PLLA: poly L-lactide. ASD: atrial septal defect. HE: haematoxylin-eosin. IHC: immunohistochemistry

After the IHC staining and HE staining, at 3 months (Fig. 9A), there were a number of fibroblasts and loose collagen fibers around the PLLA filaments (green arrow), and with a small number of inflammatory cells and a moderate number of foreign body giant cells (blue arrow), and there were clear boundaries between PLLA filaments and surrounding tissues. However, at 6 months (Fig. 9B), the inflammatory response significantly decreased compared to 3 months, with a large number of inflammatory cells deposited around the PLLA filaments. The PLLA filaments began to disintegrate but were still in good shape. At 12 and 24 months (Fig. 9 C,D), the inflammatory response significantly decreased. The PLLA filaments (green arrows) are surrounded by a large number of fibroblasts and abundant collagen fibrillation (black arrows), with a small number of inflammatory cells and foreign body giant cells (blue arrows). Most of the PLLA filaments had disintegrated into pieces. In addition, histopathological observation showed that there were no anomalies in the lung, liver, spleen, or kidney, and no infarction too.

### Scanning electron microscopic observation for endothelialization

**Fig. 10:**
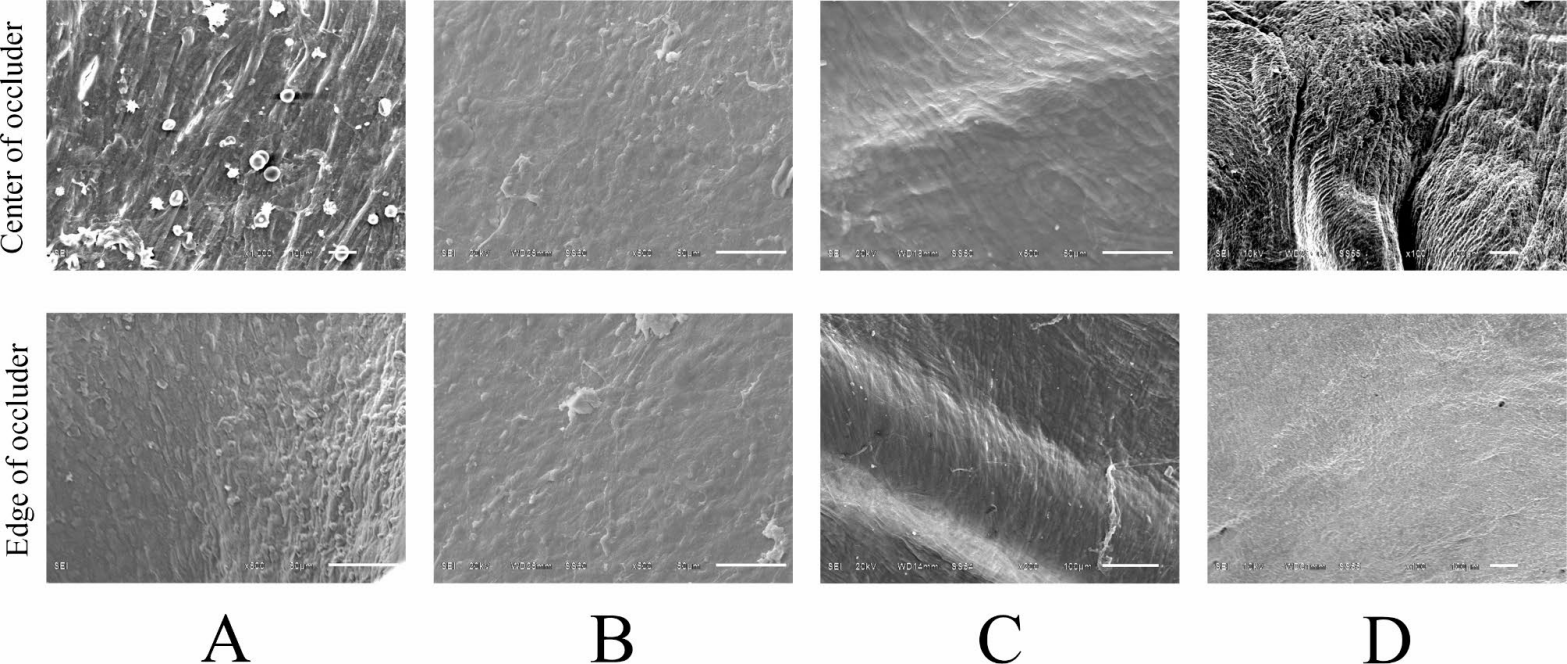
The center and edge parts of PLLA ASD occluders characterized by scanning electron microscopy (SEM) at the indicated time points of 3 (**A**), 6 (**B**), 12 (**C**), 24 months (**D**) after implantation. PLLA: poly L-lactide. ASD: atrial septal defect

At 3 months (Fig. 10A), the PLLA ASD occluders were covered by a layer of endothelial cells, but were relatively sparse. At 6 months (Fig. 10B), endothelial cells have become denser, and there was fibrous connective tissue among the endothelial cells. At 12 months (Fig. 10C), the implantation site was fully covered by a tight layer of endothelial tissue. The implantation site was also completely covered by a tight layer of endothelial tissue after 24 months (Fig. 10D). During the whole process, the edge and center of the occluders were completely covered by dense endothelial cells without any differences in morphology. This may indicate that the endothelial cells were formed at the same time both in the edge and center of the occluders.

## Discussion

With the development of interventional and cardiac catheterization techniques, interventional therapy has become the preferred treatment for ASD patients. Since the great invention of Amplatzer’s double-disc Nitinol occluder more than 20 years ago, most clinically available occluders have been made of Nitinol due to its high elasticity, namely NiTi alloy, etc. [13]. However, Nitinol occlusions still have some limitations and are associated with many complications, such as erosion or perforation of the device, delayed endothelialization, thrombosis, severe valve damage, hemolysis, nickel allergy, and even aorta-atrial fistula [14–16]. The ideal occluders would be absorbable frames that act as temporary “Bridges” and guide endogenic self-growing tissue to cover the occluders along the occluders’ edges to the defects beneath. In the end, the occluders could be absorbed gradually, leaving only the “self-growing” tissue behind, which reduced future complications and obstruction of transatrial complete healing and septal access [17]. PLLA is a semi-crystalline polymer with high tensile strength, good flexibility, good heat resistance, good thermal stability and other good processing properties and mechanical properties. In the human body, PLLA could eventually be degraded into carbon dioxide and water with bioabsorbability and biocompatibility. So far, they were used in a variety of medical devices such as absorbable sutures and prosthetic devices [18, 19]. In this study, PLLA was used as material to design and optimize the PLA ASD occluders.

Subcutaneous implantation is the most common method for measuring the histocompatibility of implant materials [20]. First, the material samples were implanted into animal subcutaneous or muscle tissue. After a certain period of time, the histological toxicity of the materials was evaluated according to the tissue reaction, and the absorption and metabolism processes of materials were observed in vivo. In this study, the evaluation method was upgraded by embedding material samples into animal vessels for the first time, which could simulate the more accurate application environment of an occluder to obtain more accurate information. To be wrapped into tissues is a milder form of the organism’s response to foreign material, including early inflammatory response and subsequent fibrogenic response. The formation of a fibrocystic wall is a pathological process caused by the body to wrap the foreign material [21]. The duration and the degree of reaction of this process are often related to the nature of the material itself, so that could better reflect the biocompatibility of the material [22]. This study showed that PLLA began to absorb water from surrounding tissues for hydration with the increase in implantation time. Then the polymer was depolymerized by hydrolysis, and the molecular chain was broken, which was manifested as the reduction of molecular weight. When PLLA were reduced into the low molecular polymers, their morphology began to gradually lose, and the upper yield strength and tensile strength decreased. Protofilar fragmentation is a major factor leading to the inflammatory response [23]. In this study, PLLA protofibrils were biodegraded at 6 months after implantation, the inflammatory response was rated as grade two, and the lumen was rated as grade one.

The vascular implantation experiment showed that PLLA occluders had good biocompatibility, so this paper further discussed their safety and practicability in animal models of ASD. The non-optimized ASD devices developed severe inflammation around the device at 3 and 6 months after implantation, although that did not harm major organs [24]. Here, we optimized the PLLA ASD occluder by reducing the number of PLLA screens while maintaining tensile strength to reduce inflammation and potential risks. In this study, HE staining showed that the inflammatory cells around the PLLA ASD occluder were slightly prominent at 3 months, and the inflammatory response was significantly reduced at 12 and 24 months.

Biodegradable occluders for ASD treatment should be required two specific functions: endothelialization and biodegradability. There was a risk of residual shunting if the biodegradable occluder has degraded and deformed before complete endothelialization [25]. Therefore, it is necessary to promote complete endothelialization before biodegradation. Although it has been reported that most ASD occluders were fully endothelialized within 3 months in animal studies, there have been few reports in longer-term studies. In this study, ASD animal models were observed and followed for up to 24 months. At 3 months after implantation, the edges of the occluders were completely covered by dense endothelial cells, and so were the center of the occluders. There was no difference in the morphology of endothelial cells between the edge and the center. At 24 months after implantation, the occluder remained in a stable endothelialization state, with consistent endothelialization at the edges and center of the device. Reports of the degradation of PLLA may be needed about 2–3 years [26, 27]. In this study, gross anatomy and histological evaluation showed that PLLA occluder remained stable in vivo during 24 months of follow-up. Therefore, this result meets the criteria for degradation of the PLLA device after complete endothelization.

## Data Availability

The datasets used and/or analysed during the current study are available from the corresponding author on reasonable request.

## References

[1] Bissessor N (2015). Current perspectives in percutaneous atrial septal defect closure devices. Med Devices (Auckl).

[2] Olasinska-Wisniewska A, Grygier M (2019). Antithrombotic/Antiplatelet treatment in Transcatheter Structural Cardiac Interventions-PFO/ASD/LAA occluder and Interatrial Shunt Devices. Front Cardiovasc Med.

[3] Lu W, Ouyang W, Wang S (2018). A novel totally biodegradable device for effective atrial septal defect closure: a 2-year study in sheep. J Interv Cardiol.

[4] Shi D, Kang Y, Zhang G (2019). Biodegradable atrial septal defect occluders: a current review. Acta Biomater.

[5] Lu A, Petit E, Jelonek K (2020). Self-assembled micelles prepared from bio-based hydroxypropyl methyl cellulose and polylactide amphiphilic block copolymers for anti-tumor drug release. Int J Biol Macromol.

[6] Cao S, Shao J, Xia Y (2019). Molecular Programming of Biodegradable Nanoworms via Ionically Induced morphology switch toward asymmetric therapeutic carriers. Small.

[7] Evgenidou E, Ofrydopoulou A, Malesic-Eleftheriadou N (2020). New insights into transformation pathways of a mixture of cytostatic drugs using Polyester-TiO2 films: identification of intermediates and toxicity assessment. Sci Total Environ.

[8] Pacharra S, Ortiz R, McMahon S (2019). Surface patterning of a novel PEG-functionalized poly-l-lactide polymer to improve its biocompatibility: applications to bioresorbable vascular stents. J Biomed Mater Res B Appl Biomater.

[9] Liang X, Gao J, Xu W (2019). Structural mechanics of 3D-printed poly(lactic acid) scaffolds with tetragonal, hexagonal and wheel-like designs. Biofabrication.

[10] Vahl TP, Gasior P, Gongora CA (2016). Four-year polymer biocompatibility and vascular healing profile of a novel ultrahigh molecular weight amorphous PLLA bioresorbable vascular scaffold: an OCT study in healthy porcine coronary arteries. EuroIntervention.

[11] Li BN, Xie YM, Xie ZF, Chen XM, Zhang G, Zhang DY, Liu XD, Zhang ZW (2019). Study of biodegradable occluder of atrial septal defect in a porcine model. Catheter Cardiovasc Interv.

[12] Xie ZF, Wang SS, Zhang ZW, Zhuang J, Liu XD, Chen XM, Zhang G, Zhang DA, Novel-Design (2016). Poly-L-Lactic acid biodegradable device for Closure of Atrial Septal defect: long-term results in Swine. Cardiology.

[13] Masura J, Gavora P, Formanek A, Hijazi ZM (1997). Transcatheter closure of secundum atrial septal defects using the new self-centering amplatzer septal occluder: initial human experience. Cathet Cardiovasc Diagn.

[14] Pranata R, Yonas E, Deka H, Vania R, July J (2020). Stent-assisted coiling of intracranial Aneurysms using a Nitinol-Based stent (Neuroform Atlas): a systematic review and Meta-analysis. Cardiovasc Intervent Radiol.

[15] Dhondt E, Vanlangenhove P, De Man M, Huyck L, Defreyne L (2020). No advantage of expanded polytetrafluoroethylene and Fluorinated Ethylene propylene-covered stents over uncovered Nitinol Stents for Percutaneous palliation of malignant infrahilar biliary obstruction: results of a single-center prospective randomized trial. J Vasc Interv Radiol.

[16] Lin C, Liu L, Liu Y, Leng J (2021). Recent developments in next-generation occlusion devices. Acta Biomater.

[17] Esmaeili A, Behnke-Hall K, Schrewe R, Schranz D (2019). Percutaneous closure of perimembranous ventricular septal defects utilizing almost ideal Amplatzer Duct Occluder II: why limitation in sizes?. Congenit Heart Dis.

[18] Fabi SG, Weiss R, Weinkle SH (2021). Absorbable suspension sutures: recommendations for Use in a Multimodal Nonsurgical Approach to Facial Rejuvenation. J Drugs Dermatol.

[19] Hasan A, Soliman S, El Hajj F, Tseng YT, Yalcin HC, Marei HE (2018). Fabrication and in Vitro characterization of a tissue Engineered PCL-PLLA Heart Valve. Sci Rep.

[20] Wu WY, Li BW, Liu YH, Wang XZ (2020). Beijing Da Xue Xue Bao Yi Xue Ban.

[21] Kian LK, Saba N, Jawaid M, Sultan MTH (2019). A review on processing techniques of bast fibers nanocellulose and its polylactic acid (PLA) nanocomposites. Int J Biol Macromol.

[22] Wu J, Gui X, Jiang H (2020). Zhongguo Xiu Fu Chong Jian Wai Ke Za Zhi.

[23] Oberhauser JP, Hossainy S, Rapoza RJ. Design principles and performance of bioresorbable polymeric vascular scaffolds. EuroIntervention. 2009;5 Suppl F:F15–F22. 10.4244/EIJV5IFA3 22100671

[24] Zhu YF, Huang XM, Cao J (2012). Animal experimental study of the fully biodegradable atrial septal defect (ASD) occluder. J Biomed Biotechnol.

[25] Li BN, Xie YM, Xie ZF (2019). Study of biodegradable occluder of atrial septal defect in a porcine model. Catheter Cardiovasc Interv.

[26] Oyama HT, Tanishima D, Ogawa R (2017). Biologically safe poly(l-lactic acid) blends with tunable degradation rate: microstructure, degradation mechanism, and Mechanical Properties. Biomacromolecules.

[27] Lin S, Dong P, Zhou C (2020). Degradation modeling of poly-l-lactide acid (PLLA) bioresorbable vascular scaffold within a coronary artery. Nanotechnol Rev.

